# Low Intrahost and Interhost Genetic Diversity of *Carnivore Protoparvovirus 1* in Domestic Cats during a Feline Panleukopenia Outbreak

**DOI:** 10.3390/v14071412

**Published:** 2022-06-28

**Authors:** Xiuwan Wang, Maura Carrai, Kate Van Brussel, Shuo Feng, Julia A. Beatty, Mang Shi, Edward C. Holmes, Jun Li, Vanessa R. Barrs

**Affiliations:** 1 City University of Hong Kong Shenzhen Research Institute, Shenzhen 518057, China; xiuwan.wang@my.cityu.edu.hk (X.W.); shuofeng8-c@my.cityu.edu.hk (S.F.); jun.li@cityu.edu.hk (J.L.); 2 Department of Infectious Diseases and Public Health, Jockey Club College of Veterinary Medicine and Life Sciences, City University of Hong Kong, Hong Kong, China; 3 Department of Veterinary Clinical Sciences, Jockey Club College of Veterinary Medicine and Life Sciences, City University of Hong Kong, Hong Kong, China; mcarrai@cityu.edu.hk (M.C.); julia.beatty@cityu.edu.hk (J.A.B.); 4 Centre for Animal Health and Welfare, City University of Hong Kong, Hong Kong, China; 5 School of Veterinary Science, Faculty of Science, University of Sydney, Sydney, NSW 2006, Australia; kate.vanbrussel@sydney.edu.au; 6 School of Life and Environmental Sciences and School of Medical Sciences, Sydney Institute for Infectious Diseases, University of Sydney, Sydney, NSW 2006, Australia; edward.holmes@sydney.edu.au; 7 School of Medicine, Sun Yat-sen University, Guangzhou 510275, China; shim23@mail.sysu.edu.cn

**Keywords:** feline panleukopenia, feline parvovirus, metagenomics, diversity, intrahost

## Abstract

Feline panleukopenia (FPL), a highly contagious and frequently fatal disease of cats, is caused by Feline parvovirus (FPV) and Canine parvovirus (CPV). We characterised the diversity of these *Carnivore protoparvovirus 1* variants in 18 faecal samples collected from domestic cats with FPL during an outbreak, using targeted parvoviral DNA metagenomics to a mean depth of >10,000 × coverage per site. All samples comprised FPV alone. Compared with the reference FPV genome, isolated in 1967, 44 mutations were detected. Ten of these were nonsynonymous, including 9 in nonstructural genes and one in VP1/VP2 (Val232Ile), which was the only one to exhibit interhost diversity, being present in five sequences. There were five other polymorphic nucleotide positions, all with synonymous mutations. Intrahost diversity at all polymorphic positions was low, with subconsensus variant frequencies (SVF) of <1% except for two positions (2108 and 3208) in two samples with SVF of 1.1–1.3%. Intrahost nucleotide diversity was measured across the whole genome (0.7–1.5%) and for each gene and was highest in the NS2 gene of four samples (1.2–1.9%). Overall, intrahost viral genetic diversity was limited and most mutations observed were synonymous, indicative of a low background mutation rate and strong selective constraints.

## 1. Introduction

Feline panleukopenia (FPL) is a highly contagious and often fatal disease of cats caused by Feline parvovirus (FPV), a small nonenveloped single-stranded DNA virus in the species *Carnivore protoparvovirus 1* (genus *Protoparvovirus*, family *Parvoviridae*) that has circulated among felid hosts for approximately 100 years [1]. FPL is characterised by severe acute enteritis and sepsis [2]. In Australia, FPL re-emerged as a major cause of mortality in shelter-housed cats between 2014 and 2018, in association with low vaccination rates [3,4]. Canine parvovirus (CPV) is a closely related variant of *Carnivore protoparvovirus 1* that emerged in domestic dogs in the mid-to-late 1970s and subsequently acquired the feline host range. CPV has been reported to cause FPL in cats [5,6,7,8]. 

When it first emerged, CPV was unable to bind the feline transferrin receptor (fTfR), which is required to initiate viral infection of the host [9]. Four mutations in the VP2 of CPV (L87M, I101T, A300G, and D305Y) in an antigenic variant (termed CPV-2a) that evolved around 1980 enabled CPV to infect and replicate in cats [10,11]. All currently circulating CPVs, including CPV-2b (VP2 N426D) [12] and CPV-2c (VP2 D426E) [13], can infect cats and cause FPL, as evidenced by naturally occurring and experimental infections [5,6,7,8]. 

Mixed infections of FPV and CPV have been occasionally detected in both healthy and diseased cats using conventional VP2 PCR and sequencing. Such coinfections have been suggested to be a source of complexity and diversity of parvoviral genetic variation [14,15,16,17]. The rapid development of next-generation sequencing (NGS) has facilitated research in viral diversity through amplicon sequencing [18,19,20]. Here, we applied a metagenomics method combining NGS with targeted hybridisation probes to capture parvoviral DNA present in faecal samples from cats with FPL, obtained during the re-emergent Australian FPL outbreak [3]. Our aims were to determine whether CPV coinfections were present in FPV-infected cats with FPL and to reveal the extent and pattern of intra- and interhost *Carnivore protoparvovirus* *1* genetic diversity. 

## 2. Materials and Methods

### 2.1. Sample Collection

Eighteen faecal samples collected from cats with FPL from multiple shelters in 2016–2017 during a re-emergent epizootic of FPL in Sydney, New South Wales, Australia, were included in the study (Supplementary Materials Table S1). The presence of *Carnivore Protoparvovirus 1*, specifically FPV, had been confirmed by PCR of the partial capsid VP-2 gene and Sanger sequencing [3]. 

### 2.2. DNA Extraction, Baiting and Sequencing

Viral nucleic acids (RNA and DNA) were extracted using the QIAamp Viral RNA Mini Kit (Qiagen, Hilden, Germany) after enrichment according to a published protocol [21] with minor modifications [22] and split into two aliquots. One aliquot was used for RNA sequencing in another study [23]. For the other aliquot, viral genomic DNA was subjected to whole genome amplification using the Whole Genome Amplification Kit (WGA2) (Sigma-Aldrich, St. Louis, MO, USA) and purified using the GenElute PCR Clean-Up Kit (Sigma-Aldrich, St. Louis, MO, USA). Amplified DNA was quantified by Qubit 2.0 fluorometer. DNA libraries were created using the Nextera XT Library Preparation Kit (Illumina, San Diego, CA, USA) with no size selection and enriched for *Carnivore protoparvovirus 1* using customised, hybridisation-based target capture kits (myBaits, Arbor Biosciences, Ann Arbor, MI, USA). A total of 12,417 biotinylated RNA probe baits were designed with 80 bp with 3 × tiling, based on all *Carnivore protoparvovirus 1* sequences downloaded from NCBI GenBank targeting the VP2 gene. The enrichment method followed the manufacturer’s instructions with minor modifications, including a pre-treatment phase to deplete any remaining streptavidin in the DNA libraries. One set of capture reactions was performed using a hybridisation temperature of 62 °C for 16 h. All PCR amplifications were carried out using KAPA Hi HotStart Mix (Kapa Biosystems, Cape Town, South Africa) with the “reamp” primers IS5_reamp.P5AATGATACGGCGACCACCGA and S6_reamp.P7CAAGCAGAAGACGGCATACGA [24], followed by purification with the GenElute PCR Clean-up kit (Sigma-Aldrich, St. Louis, MO, USA). The enriched target DNA of two libraries with low DNA concentrations was sequenced at the AGRF (Melbourne, Australia) using an Illumina NextSeq500 platform with NextSeq500 sequencing-300 cycles System midi-Output Kit (Illumina, San Diego, CA, USA). The remaining 16 samples were sequenced on the same platform using the NextSeq500 System High-Output Kit (Illumina, San Diego, CA, USA) with a final output of 32.5–39 Gb and 100–120 Gb, respectively. 

### 2.3. Detection and Characterisation of FPV and CPV 

We mapped all raw reads to the cat reference genome (Felis catus 9.0 assembly, GenBank Assembly ID GCA_000181335.4) using BWA version 0.7.17 [25] and removed those with more than 95% mapping coverage. We then further processed the raw reads for quality control with the following procedures [26,27] (available at https://github.com/TingtZHENG/metagenomics/blob/master/scripts/fqc.pl (accessed on 1 June 2022)): (i) remove Illumina primers, adaptors, and linker sequences; (ii) remove paired-ends reads with 25 bp consecutively exact match from both ends to eliminate PCR duplicates; (iii) remove terminal regions with continuous Phred-based quality of less than 20. Reads were then mapped to the reference FPV sequence (FPV-3, GenBank accession no. EU659111.1) using BWA version 0.7.17 [25]. Reads with over 90% mapping coverage were then extracted. Sequencing depth and error rate across the entire genome were also determined.

We similarly mapped all the extracted reads against the FPV-3 reference genome using BWA version 0.7.17 [25]. Next, we converted BWA sam output to the bam format with SAMtools version 1.9 [28]. Based on the bam results for each sample, variant calling was performed by BCFtools version 1.9 [28]. First, BCFtools mpileup was used to generate Variant Call Format (VCF) files from bam results. Subsequently, the BCFtools call was utilised with a smaller mutation rate (0.01%) to obtain stricter calls for single nucleotide variants (SNV). Only SNVs with a QUAL (Phred-scaled probability) > 20 were retained. The residues at 26 amino acid positions (Supplementary Materials File S1) in the VP2 gene were used to characterise SNVs as FPV or CPV [29,30,31]. 

### 2.4. Evolutionary Analysis

The Metagenomic Intraspecies Diversity Analysis System (MIDAS) [32] was used to identify the majority consensus sequence for each sample. Using the FPV reference genome (FPV-3.us.67; GenBank accession no. EU659111), MIDAS used Bowtie2 [33] to globally align reads against the genome database. Mapped reads were used to determine the consensus sequences. 

A separate study was conducted to characterise the faecal viromes of 17 of the same 18 faecal samples we analysed using metatranscriptomics without virion enrichment or baits [23]. The metatranscriptomics parvoviral data set was used to compare the accuracy and sensitivity of our ability to detect parvoviruses and SNPs in these 18 samples (Supplementary Materials File S3). In comparison with the FPV reference genome, we investigated the distribution of mutations for each sample in this metatranscriptomics parvoviral data set and our 18 consensus sequences using MUSCLE [34] in the MEGAX version 10.1.8 analytical package [35].

We used phylogenetic analysis to reveal the evolutionary relationships between the FPV sequences obtained in this study and the reference sequences at the full-genome and individual gene levels. A total of 33 whole-genome FPV sequences (Supplementary Materials Table S2) were downloaded from GenBank after excluding highly identical or identical sequences from the same geographic region and year. In addition, we chose four CPV sequences to represent CPV-2, CPV-2a, CPV-2b, and CPV-2c. Prior to phylogenetic analysis, all 55 sequences (37 sequences from NCBI and 18 sequences from this study) were screened for recombination using the Recombination Detection Program 4 (RDP4) [36], employing the RDP, GENECOV, and Bootscan programs and requiring a *p*-value of ≥0.05 and a consensus recombination score > 0.6. 

Evolutionary relationships were determined by aligning the full genome, NS1 (58 sequences from NCBI) and VP2 (62 sequences from NCBI) nucleotide sequences (Supplementary Materials Table S2) retrieved from NCBI GenBank with MAFFT version 7 employing the E-INS-I algorithm [37]. Phylogenetic trees were inferred using the maximum likelihood approach in IQ-TREE [38] and the ModelFinder program [39] to the best-fit model of nucleotide substitution. Nodal support was accessed using the SH-like approximate likelihood ratio test and ultrafast bootstrap approximation (SH-aLRT/UFBoot) and 1000 replicates [40]. 

### 2.5. Nucleotide Diversity Analysis

We used MIDAS [31] to estimate the most abundant minor variant frequency and then calculated intrahost sample nucleotide diversity (pi diversity, π) for each site. In addition, average nucleotide diversity was calculated at the individual gene and full-genome levels.

## 3. Results

### 3.1. Overview 

**Figure 1:**
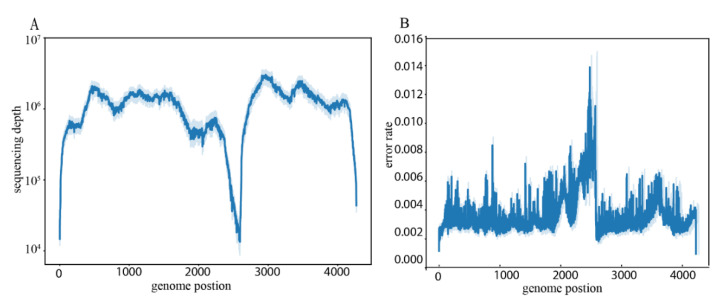
Overall description of the sequencing information in this study (mean lines with confidence interval bands). Dark blue represents the mean line, and light blue areas represent confidence intervals. (**A**) Sequencing depth along the genome coordinate. (**B**) Sequencing error rate along the genome coordinate.

A total of 18 sequencing libraries were generated for the analysis of intra- and interhost feline parvovirus diversity. Read files generated by the sequencer provided near-complete genome coverage, covering all major open reading frames. We calculated confidence intervals for the mean of the sequencing depth, obtaining over 10,000 × coverage per site (Figure 1A) after the application of the quality control for raw reads. A short region (site 2470–2620, length: 150 nucleotides (nt)) flanked by a poly(A)- and G-rich sequence region near the beginning of the VP2 gene showed a lower sequencing depth (Figure 1A) and higher error rate (Figure 1B) when compared with other regions. We considered this short region and two regions at the beginning (site 1–10) and end (site 4260–4269) as three low coverage regions (LCRs) because of insufficient sequencing. Although LCRs did not influence calling the consensus sequences, they did affect the accuracy and sensitivity of variant frequency estimations. Thus, LCRs were excluded when determining intrahost diversity. In the following analyses, genome nucleotide numbering begins at the first position of the NS1/NS2 gene coding region, and amino acid numbering starts at the first methionine for each respective gene.

### 3.2. Characterization of Carnivore Protoparvovirus 1 and Mutation Analysis

Using BCF tools, the number of SNVs detected for each sample in comparison to the reference genome ranged from 39 to 41 (Supplementary Materials File S2). All SNVs had a high Phred-scaled probability (130–218). All 18 samples had 39 identical SNVs compared with the reference FPV genome (EU659111). In addition, five other nucleotide positions were associated with SNVs in some samples. None of the SNVs detected in any sample were CPV-related single nucleotide polymorphism (SNP) markers. Thus, there was no evidence of CPV coinfection in any of the 18 samples. An FPV consensus sequence was determined for each sample (GenBank accession numbers MZ742166—MZ742180, Supplementary Materials Table S1) and compared with the FPV reference genome, spanning both the NS and VP coding regions (length: 4269 bp, hereafter referred to as the full genome). A total of 44 nucleotide positions (with LCR regions removed) were found to have mutations among the 18 FPV consensus sequences. All 44 positions shared the same mutations for each sample in the metatranscriptomics parvoviral data set as the 18 consensus sequences generated here (Supplementary Materials Figure S1 and File S3).

### 3.3. Interhost FPV Diversity

**Figure 2:**
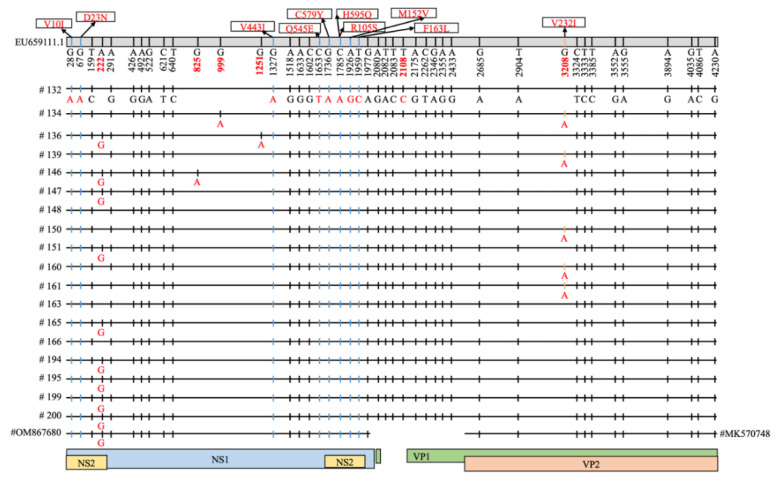
Mutations in the 18 FPV consensus sequences in this study and closely related VP2 (#MK570748) and NS1 (#OM867680) gene sequences in comparison with the FPV reference genome (EU659111). The nucleotide position is indicated in the bar at the top of the figure, together with changes in amino acid sequence, and the character state of individual nucleotides is indicated below for each consensus sequence. Synonymous mutations are indicated by black vertical bars, and nonsynonymous mutations are indicated by blue or orange vertical bars, corresponding to the relevant gene segment indicated at the bottom of the figure. Nucleotides of other sequences that are the same as for sample #132 are indicated by a vertical bar. A total of 10 nonsynonymous mutations (9 nucleotide positions were involved) were identified (NS1/NS2: Val10Ile, Asp23Asn; NS1: Val443Ile, Gln545Glu, Cys579Tyr, His595Gln; NS2: Arg105Ser, Met152Val, Phe163Leu; VP1/VP2: Val232Ile). The six nucleotide diversity positions (222, 825, 999, 1251, 2108, and 3208) are indicated by red font on the genome bar at the top of the figure.

Of the 10 nonsynonymous mutations identified compared with the FPV reference strain (Supplementary Materials Figure S1 and Table S3), only one—Val232Ile—exhibited interhost diversity among the 18 FPV sequences, being present in five sequences (Figure 2). In addition, five other polymorphic nucleotide positions were identified, all of which were synonymous substitutions (Figure 2).

### 3.4. Evolutionary Analysis

**Figure 3:**
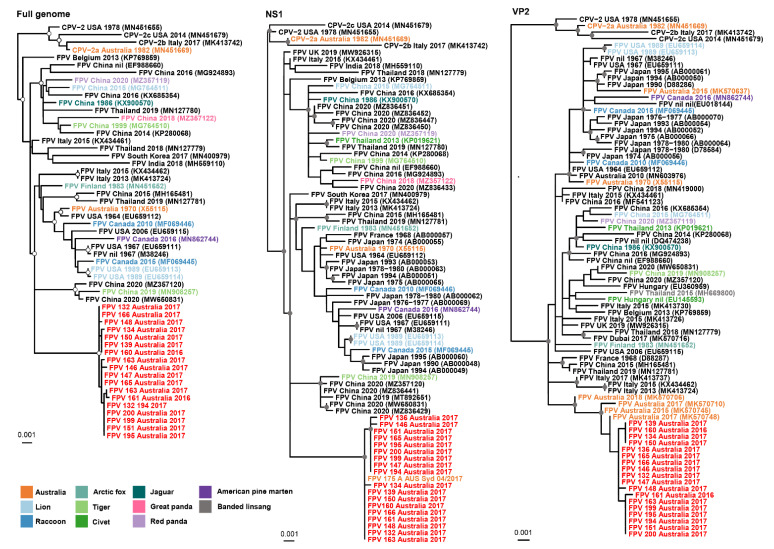
Phylogenetic relationships of the 18 FPV sequences described in this study and those retrieved from NCBI GenBank. All maximum likelihood trees are midpoint rooted for clarity, and sequences described in this study are highlighted in red and other Australian sequences in orange. Nodal support greater than 80% SH-aLRT and 95% UFBoot are represented by a grey circle. Sequences are designated in the phylogenetic trees by virus type (FPV or CPV), followed by the country year of isolation, and finally, accession number. The NS1 sequences for the Australian strains MK570748, MK570637, MK570710, and MK570706 are unavailable and therefore missing from both the full genome and NS1 phylogenies.

We scanned all 55 whole-genome sequences of FPV (37 from GenBank and 18 consensus sequences in this study) with RDP4 and detected no recombinant genomes. The FPV sequences from this study formed a distinct clade in the full genome, NS1 and VP2 phylogenies, supported by strong support values in the full genome and NS1 trees, although the positioning of the sequences within the clade has little resolution (Figure 3). Australian FPV sequences obtained by our research group for a previous study are most closely related to those presented here, as is particularly clear in the VP2 phylogeny [3]. 

### 3.5. Intrahost Analyses

**Figure 4:**
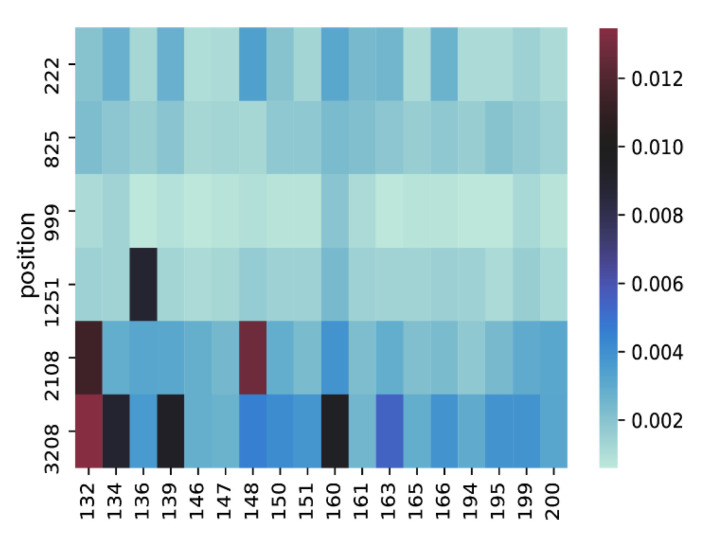
Heatmap of the subconsensus variant frequencies (SVF) in six diversity positions. SVF are displayed as colours ranging from blue to dark red. SVF was <1% in six positions in all samples, except for two positions in two samples (2108: #132 (SVF = 1.1%), #148 (SVF = 1.3%); 3208: #132 (SVF = 1.3%)).

We next measured the subconsensus variant frequency (SVF) in each sequence, itself reflecting the most abundant minor variant frequency for each site. A low SVF implies that the major variant is conserved. Of the six polymorphic nucleotide positions identified among the 18 FPV sequences, all had low SVF (< 1%) except for two positions in two samples, which had SVF > 1%, varied from 1.1% to 1.3% (Figure 4).

**Figure 5:**
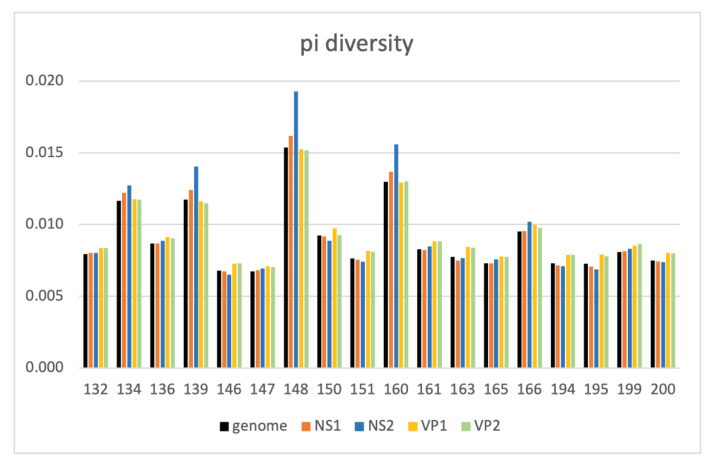
Nucleotide diversity (π) across the whole genome and per gene (NS1, NS2, VP1, and VP2). The whole genome within-sample π diversity varied between 0.7% and 1.5%. Four samples (#134, #139, #148, and #160) had a pi diversity >1%.

Nucleotide diversity, defined as the average number of nucleotide differences between the same regions in a sample (population) (i.e., π or pi diversity), was used to measure the degree of polymorphism in a sample. For each sample, we quantified π along the FPV genome (excluding the LCRs) and for each gene (Supplementary Materials Figure S2). The whole genome within-sample π diversity varied between 0.7% and 1.5%. A pi diversity of >1% was observed in four samples (#134, #139, #148, and #160). The highest pi diversity (1.9%) was observed in the NS2 gene for sample #148 (Figure 5, Supplementary Materials Table S4).

## 4. Discussion

There are few studies on the intrahost and interhost diversity of FPV. Here, using a fine-scale metagenomics approach, we identified 44 mutations (10 nonsynonymous) in Australian FPV strains collected in 2016/2017 compared with the reference strain (FPV-3) isolated in 1967. By contrast, an earlier study examined NS1 clones (7 to 40) and VP2 clones (5 to 40) from six strains of FPV in various feline tissues collected over a 43-year period and found 33 mutations (six nonsynonymous) compared with FPV-4, isolated in 1963 [41]. The limited FPV genetic diversity observed at both the inter- and intra- host levels, largely comprising synonymous mutations, is strongly suggestive of a low background mutation rate and the presence of strong selective constraints against amino acid variation.

Most studies on *Carnivore protoparvovirus 1* evolution have focused on the VP2 gene because of its role in host range determination, whereas mutations in the NS genes have been investigated infrequently. Notably, we identified only one nonsynonymous mutation in FPV genomes in this study in the VP2 coding region. This mutation (Val232Ile) has been detected previously on several occasions [3,42,43]. In contrast to previous work, 9 of the 10 nonsynonymous mutations detected here were in the NS coding region. Of the six nonsynonymous mutations in FPV strains analysed by Hoelzer et al. (2008), three were in NS1 and three were in the VP genes (2 in VP1, 1 in VP2).

Two NS1 gene mutations we detected are novel (Val10Ile and Cys579Tyr), while the other four have been detected previously (Asp23Asn, Val443Ile, Gln545Glu, and His595Gln). The three nonsynonymous mutations (Arg105Ser, Met152Val and Phe163Leu) we found in the NS2 gene have been detected previously in FPVs from Italy (Arg105Ser), Europe, and China (Met152Val), and the Phe163Leu substitution is common globally [44]. For CPV, although most naturally occurring mutations are synonymous and involve the VP2 gene, several have also arisen and become widespread in the NS genes, including the Met152Val substitution detected in our FPV genomes [30,31]. Parvoviral NS proteins interact with the cellular proteins of the host, so it is possible that selection pressures differ for cats compared with dogs, although the phenotypic effects of NS gene mutations in CPV and FPV are poorly understood. 

For phylogenetic trees based on the whole genome, NS1 and VP2, strains were mainly clustered by country and year of isolation. The 18 FPV sequences we examined were clustered closely in a single clade (Figure 4) and were most closely related to Australian viruses from the same region over a similar time frame. Because there are more polymorphic positions in NS1 than in VP2, there was a bootstrap-supported subclade of FPVs in this study in the phylogenetic tree for NS1 but not for VP2. In the whole genome and NS1 phylogenies, the viruses most closely related to the Australian FPVs were from a tiger and domestic cats in 2019 and 2020 from China that fell in a clade with high bootstrap support. In the VP2 tree, apart from an Australian virus collected from a domestic cat in 2018, the most closely related viruses were from domestic cats in Italy. 

Since cats are susceptible to both FPV and CPV and given the resilience of these viruses, which can withstand harsh environmental conditions for at least 12 months [2], individual cats could be coinfected with multiple *Carnivore protoparvovirus 1* strains, facilitating recombination. However, using targeted hybridised baits to capture parvovirus DNA, we found no evidence of FPV/CPV coinfections in the faeces of any cats with FPL sampled during an outbreak, although the sample size was small.

Although some evidence suggests that CPV is displacing FPV among wild carnivores [45,46], this is not the case among domestic cats, where no outbreaks of FPL have been attributed to CPV. Similarly, FPV/CPV coinfections appear to be sporadic, being detected in only a few cases by cloning and Sanger sequencing PCR products of strains with VP2 and NS1 chromatograms suggestive of mixed viral populations [14,15,17]. One cat with enteritis was coinfected with FPV, CPV-2a, and a parvovirus variant with intermediate characteristics between FPV and CPV-2a [15]. Similarly, we determined that faecal shedding of CPV is uncommon among asymptomatic shelter-housed cats [47]. 

Finally, the high genetic diversity in the NS2 gene of several samples in this study highlights the need for additional investigations to determine the frequency of diversity observed in this gene among a larger population of feline parvoviruses. 

## Data Availability

Sequencing data for 18 samples were archived in the National Center for Biotechnology Information (NCBI) Short Read Archive (SRA): PRJNA836632. Including the new 18 FPV consensus sequences, all genome sequences analysed in this study were downloaded from the NCBI GenBank database. The accession numbers are listed in Supplementary Materials Tables S1 and S2.

## Supplementary Materials

The following supporting information can be downloaded at: https://www.mdpi.com/article/10.3390/v14071412/s1, Table S1: Sample description; Table S2: Summary table of genomes used in phylogenetic analyses; Table S3: Position and translational effect of FPV nucleotide substitutions; Table S4: Nucleotide diversity (π) on whole genome and per gene (NS1, NS2, VP1, and VP2); Figure S1: Position of FPV nucleotide substitutions in 18 full genome FPV strains from this study as compared to the FPV reference strain (EU659111); Figure S2: Nucleotide diversity (π) along the genome coordinate; File S1: list of the residues at 26 amino acid positions in the VP2 gene characterised SNVs as FPV or CPV; File S2: Summary of SNVs detected in 18 samples; File S3: Comparison of the 18 consensus sequences and the metatranscriptomics parvoviral data set.
