# Supplementary material for: Low Intrahost and Interhost Genetic Diversity of Carnivore Protoparvovirus 1 in Domestic Cats during a Feline Panleukopenia Outbreak
Source: Viruses. 2022 Jun 28;14(7):1412. doi: 10.3390/v14071412 (PMC9325248; doi:10.3390/v14071412)
Supplement: Supplementary file 1 [file viruses-14-01412-s001.zip › viruses-1742016-suppl.pdf]

Supplementary Materials

Table S1. Overview description of 18 samples used in this study. All the cats were Domestic Short Hair and different features like the location of cats are indicated.

ID	Shelter	Location of Cat	GenBank Accession
132	Shelter 1	Greater Western Sydney (Kemps creek)	MZ742166
134	Privately owned	Inner Western Sydney (Croydon Park)	MZ742167
136	Shelter 2	Western Sydney (Granville)	MZ742168
139	Shelter 2	Western Sydney (Yagoona)	MZ742169
146	Veterinary Hospital	Western Sydney (Bass Hill)	MZ742170
147	Veterinary Hospital	Western Sydney (Bass Hill)	MZ742171
148	Shelter 2	Western Sydney (Yagoona)	MZ742172
150	Shelter 2	Western Sydney (Yagoona)	MZ742163
151	Shelter 2	Western Sydney (Yagoona)	MZ742164
160	Shelter 2	Western Sydney (Yagoona)	MZ742173
161	Shelter 2	Western Sydney (Yagoona)	MZ742174
163	Shelter 1	Greater Western Sydney (Kemps creek)	MZ742175
165	Shelter 1	Greater Western Sydney (Kemps creek)	MZ742176
166	Shelter 1	Greater Western Sydney (Kemps Creek)	MZ742177
194	Shelter 3	Inner Western Sydney (Concord)	MZ742178
195	Shelter 3	Inner Western Sydney (Concord)	MZ742165
199	Shelter 4	Greater Western Sydney (Blacktown)	MZ742179
200	Shelter 4	Greater Western Sydney (Blacktown)	MZ742180

Table S2. Summary table of the information about 31 FPV genomes, four CPV representative genomes, and one MEV genomes downloaded from GenBank. FPV: feline parvovirus, CPV: canine parvovirus.

Accession No.	Host	Virus	Country	Year Isolated	Region
EU659111.1	Domestic cat	FPV	USA	1967	Full genome, NS1, VP2
M38246.1	NA	FPV	USA	1967	Full genome, NS1, VP2
KP769859.1	Domestic cat	FPV	Belgium	2013	Full genome, NS1, VP2
KX900570.1	Jaguar	FPV	China	1986	Full genome, NS1, VP2
KX434462.1	Cat	FPV	Italy	2015	Full genome, NS1, VP2
MG924893.1	feline	FPV	China	2016	Full genome, NS1, VP2
MH559110.1	Domestic cat	FPV	India	2018	Full genome, NS1
MG764510.1	Panthera Tigris	FPV	China	1999	Full genome, NS1
MG764511.1	Leopard	FPV	China	2015	Full genome, NS1, VP2
KP280068.1	Cat	FPV	China	2014	Full genome, NS1, VP2
KX685354.1	Tiger	FPV	China	2016	Full genome, NS1, VP2
MN908257.1	South China tiger	FPV	China	2019	Full genome, NS1, VP2
MF069445.1	Raccoon	FPV	Canada	2015	Full genome, NS1, VP2
MF069446.1	Raccoon	FPV	Canada	2010	Full genome, NS1, VP2
MN862744.1	American-pine-marten	FPV	Canada	2016	Full genome, NS1, VP2
MN127779.1	Domestic cat	FPV	Thailand	2018	Full genome, NS1, VP2
MN127781.1	Domestic cat	FPV	Thailand	2019	Full genome, NS1, VP2
MN127780.1	Domestic cat	FPV	Thailand	2019	Full genome, NS1
EF988660.1	Cat	FPV	China	2007	Full genome, NS1, VP2
EU659112.1	Domestic cat	FPV	USA	1964	Full genome, NS1, VP2
EU659113.1	Mountain lion	FPV	USA	1989	Full genome, NS1, VP2
EU659115.1	Domestic cat	FPV	USA	2006	Full genome, NS1, VP2
MK413724.1	Cat	FPV	Italy	2013	Full genome, NS1, VP2
MN451652.1	Vulpes-lagopus	FPV	Finland	1983	Full genome, NS1, VP2
EU659114.1	lion	FPV	USA	1989	Full genome, NS1, VP2

KX434461.1	cat	FPV	Italy	2015	Full genome, NS1, VP2
MH165481.1	cat	FPV	China	2015	Full genome, NS1, VP2
MN400979.1	cat	FPV	South Korea	2017	Full genome, NS1
MW650831.1	cat	FPV	China	2020	Full genome, NS1, VP2
MZ357119.1	red panda	FPV	China	2020	Full genome, NS1, VP2
MZ357120.1	cat	FPV	China	2020	Full genome, NS1, VP2
MZ357122.1	giant panda	FPV	China	2018	Full genome, NS1
X55115.1	cat	FPV	Australia	1970	Full genome, NS1, VP2
MN451655.1	Dog	CPV-2	USA	1978	Full genome, NS1, VP2
MN451669.1	Dog	CPV-2a	Australia	1982	Full genome, NS1, VP2
MK413742.1	Dog	CPV-2b	Italy	2017	Full genome, NS1, VP2
MN451679.1	Dog	CPV-2c	USA	2014	Full genome, NS1, VP2
AB000048.1	cat	FPV	Japan	1990	NS1, VP2
AB000049.1	cat	FPV	Japan	1994	NS1
AB000051.1	cat	FPV	Japan	1994	NS1
MW926315.1	cat	FPV	UK	2019	NS1
AB000053.1	cat	FPV	Japan	1993	NS1
AB000055.1	cat	FPV	Japan	1974	NS1
AB000057.1	cat	FPV	France	1968	NS1
AB000060.1	cat	FPV	Japan	1995	NS1
KP019621.1	civet	FPV	Thailand	2013	NS1
AB000063.1	cat	FPV	Japan	1978	NS1
AB000065.1	cat	FPV	Japan	1975	NS1
AB000069.1	cat	FPV	Japan	1976	NS1
AB000062.1	cat	FPV	Japan	1978	NS1
MT892651.1	cat	FPV	China	2019	NS1
MZ836452.1	cat	FPV	China	2020	NS1
MZ836451.1	cat	FPV	China	2020	NS1
MZ836450.1	cat	FPV	China	2021	NS1
MZ836429.1	cat	FPV	China	2020	NS1
MZ836447.1	cat	FPV	China	2020	NS1
MZ836441.1	cat	FPV	China	2020	NS1
MZ836433.1	cat	FPV	China	2020	NS1
AB000048.1	cat	FPV	Japan	1990	NS1
MK570637.1	cat	FPV	Australia	2015	VP2
MK570748.1	cat	FPV	Australia	2017	VP2
MK570654.1	cat	FPV	Australia	2017	VP2
MK570710.1	cat	FPV	Australia	2017	VP2
MK570706.1	cat	FPV	Australia	2018	VP2
MK570716.1	cat	FPV	Dubai	2017	VP2
MN603976.1	cat	FPV	Australia	2010	VP2
AB000056.1	cat	FPV	Japan	1964	VP2
AB000054.1	cat	FPV	Japan	1993	VP2
AB000070.1	cat	FPV	Japan	1976	VP2
MF541123.1	cat	FPV	China	2016	VP2
AB000052.1	cat	FPV	Japan	1994	VP2
AB000064.1	cat	FPV	Japan	1978	VP2
AB000066.1	cat	FPV	Japan	1975	VP2
AB000050.1	cat	FPV	Japan	1994	VP2
AB000061.1	cat	FPV	Japan	1995	VP2
D88286.1	cat	FPV	Japan	1990	VP2
D88287.1	cat	FPV	France	1968	VP2
MK413730.1	cat	FPV	Italy	2015	VP2
D78584.1	cat	FPV	Japan	1978	VP2
MK413726.1	cat	FPV	Italy	2015	VP2
MK413737.1	cat	FPV	Italy	2017	VP2

EU145593.1	civet	FPV	Hungary	NA	VP2
MN419000.1	cat	FPV	China	2018	VP2
DQ474238.1	NA	FPV	NA	NA	VP2
EU360959.1	cat	FPV	Hungary	NA	VP2
MH669800.1	Banded linsang	FPV	Thailand	2015	VP2
EU018144.1	cat	FPV	Argentina	NA	VP2

Table S3. Position and translational effect of FPV nucleotide substitutions. Nucleotide numbering begins at the first position of the NS1/NS2 gene coding region, and amino acid numbering starts at the first methionine for each respective gene. NS1/NS2 and VP1/VP2 refers to the overlap region of these genes. Positions with inter-host diversity among the 18 samples in this study are indicated by bold font.

Nucleotide Position	Nucleotide Change	Translational Effect	Gene Region
28	G -> A	Val10Ile	NS1/NS2
67	G -> A	Asp23Asn	
159	T -> C	Synonymous	
**222**	A -> G	Synonymous	
291	A -> G	Synonymous	NS1
426	A -> G	Synonymous	
492	A -> G	Synonymous	
522	G -> A	Synonymous	
621	C -> T	Synonymous	
640	T -> C	Synonymous	
**825**	G -> A	Synonymous	
**999**	G -> A	Synonymous	
**1251**	G -> A	Synonymous	
1327	G -> A	Val443Ile	
1518	A -> G	Synonymous	
1602	A -> G	Synonymous	
1633	C -> G	Synonymous	
1653	C -> T	Gln545Glu	
1736	G -> A	NS1 Cys579Tyr<br>NS2 Synonymous	NS1/NS2
1785	C -> A	NS1 His595Gln<br>NS2 Arg105Ser	
1926	A -> G	NS1 Synonymous<br>NS2 Met152Val	
1959	T -> C	NS1 Synonymous<br>NS2 Phe163Leu	
1977	G -> A	Synonymous	NS1
2080	A -> G	VP1 Intron	
2082	T -> A	VP1 Intron	
2083	T -> C	VP1 Intron	
**2108**	T -> C	VP1 Intron	
2175	A -> G	Synonymous	VP1
2262	C -> T	Synonymous	
2346	G -> A	Synonymous	
2355	A -> G	Synonymous	
2433	A -> G	Synonymous	
2685	G -> A	Synonymous	VP1/VP2
2904	T -> A	Synonymous	
**3208**	G -> A	Val232Ile	
3324	C -> T	Synonymous	
3333	T -> C	Synonymous	
3385	T -> C	Synonymous	
3552	A -> G	Synonymous	

Nucleotide Position	Nucleotide Change	Translational Effect	Gene Region
3555	G -> A	Synonymous	
3894	A -> G	Synonymous	
4035	G -> A	Synonymous	
4086	T -> C	Synonymous	
4230	A -> G	Synonymous	

Table S4. Nucleotide diversity (π) on whole genome and per gene (NS1, NS2, VP1, VP2). AVG, average nucleotide diversity between the regions in a sample; SE, Standard Error; IQR, interquartile range, is the difference between Q3 and Q1.

ID	Genome AVG ± SE(IQR)	NS1 AVG ± SE(IQR)	NS2 AVG ± SE(IQR)	VP1 AVG ± SE(IQR)	VP2 AVG ± SE(IQR)
132	0.008 ± 0.012(0.004)	0.008 ± 0.008(0.005)	0.008 ± 0.008(0.005)	0.008 ± 0.015(0.004)	0.008 ± 0.016(0.004)
134	0.012 ± 0.019(0.007)	0.012 ± 0.017(0.008)	0.013 ± 0.016(0.009)	0.012 ± 0.02(0.007)	0.012 ± 0.021(0.007)
136	0.009 ± 0.013(0.005)	0.009 ± 0.01(0.005)	0.009 ± 0.01(0.006)	0.009 ± 0.015(0.005)	0.009 ± 0.016(0.005)
139	0.012 ± 0.019(0.007)	0.012 ± 0.017(0.008)	0.014 ± 0.018(0.01)	0.012 ± 0.02(0.007)	0.011 ± 0.02(0.007)
146	0.007 ± 0.011(0.004)	0.007 ± 0.006(0.004)	0.007 ± 0.006(0.004)	0.007 ± 0.014(0.003)	0.007 ± 0.014(0.003)
147	0.007 ± 0.01(0.003)	0.007 ± 0.007(0.004)	0.007 ± 0.006(0.004)	0.007 ± 0.013(0.003)	0.007 ± 0.014(0.003)
148	0.015 ± 0.026(0.01)	0.016 ± 0.027(0.011)	0.019 ± 0.035(0.013)	0.015 ± 0.025(0.009)	0.015 ± 0.026(0.009)
150	0.009 ± 0.015(0.005)	0.009 ± 0.011(0.005)	0.009 ± 0.01(0.006)	0.01 ± 0.018(0.005)	0.009 ± 0.018(0.005)
151	0.008 ± 0.012(0.004)	0.008 ± 0.007(0.004)	0.007 ± 0.007(0.004)	0.008 ± 0.015(0.004)	0.008 ± 0.016(0.003)
160	0.013 ± 0.021(0.008)	0.014 ± 0.019(0.008)	0.016 ± 0.021(0.01)	0.013 ± 0.022(0.007)	0.013 ± 0.023(0.007)
161	0.008 ± 0.013(0.004)	0.008 ± 0.008(0.004)	0.008 ± 0.009(0.005)	0.009 ± 0.016(0.004)	0.009 ± 0.017(0.004)
163	0.008 ± 0.013(0.004)	0.007 ± 0.007(0.004)	0.008 ± 0.008(0.005)	0.008 ± 0.016(0.004)	0.008 ± 0.017(0.004)
165	0.007 ± 0.012(0.004)	0.007 ± 0.007(0.004)	0.008 ± 0.008(0.005)	0.008 ± 0.015(0.003)	0.008 ± 0.016(0.003)
166	0.01 ± 0.015(0.006)	0.01 ± 0.012(0.006)	0.01 ± 0.014(0.007)	0.01 ± 0.018(0.005)	0.01 ± 0.018(0.005)
194	0.007 ± 0.011(0.004)	0.007 ± 0.007(0.004)	0.007 ± 0.007(0.004)	0.008 ± 0.014(0.004)	0.008 ± 0.015(0.004)
195	0.007 ± 0.012(0.004)	0.007 ± 0.007(0.004)	0.007 ± 0.007(0.004)	0.008 ± 0.015(0.004)	0.008 ± 0.015(0.003)
199	0.008 ± 0.012(0.004)	0.008 ± 0.008(0.004)	0.008 ± 0.008(0.005)	0.009 ± 0.015(0.004)	0.009 ± 0.015(0.004)
200	0.007 ± 0.011(0.004)	0.007 ± 0.008(0.004)	0.007 ± 0.008(0.005)	0.008 ± 0.014(0.004)	0.008 ± 0.015(0.004)

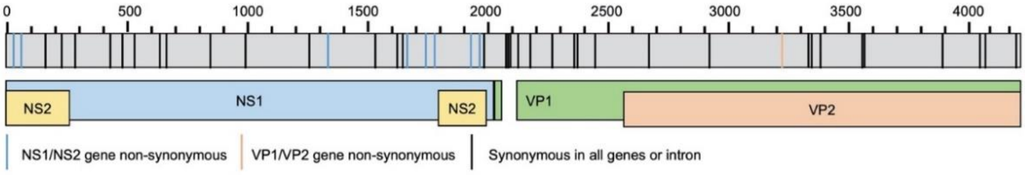

Figure S1. Position of FPV nucleotide substitutions in 18 full genome FPV strains from this study as compared to the FPV reference strain (EU659111.1). Nucleotide numbering begins at the first position of the NS1/NS2 gene coding region, and amino acid numbering starts at the first methionine for each respective gene. Synonymous changes are indicated by black vertical bars, and nonsynonymous changes are indicated by blue or orange vertical bars, while the location in the viral genome is inferred by the genome map shown below the sequences. A total of 44 positions had nucleotide substitutions among the 18 new FPV consensus sequences, including 10 non-synonymous mutations at 9 nucleotide positions.

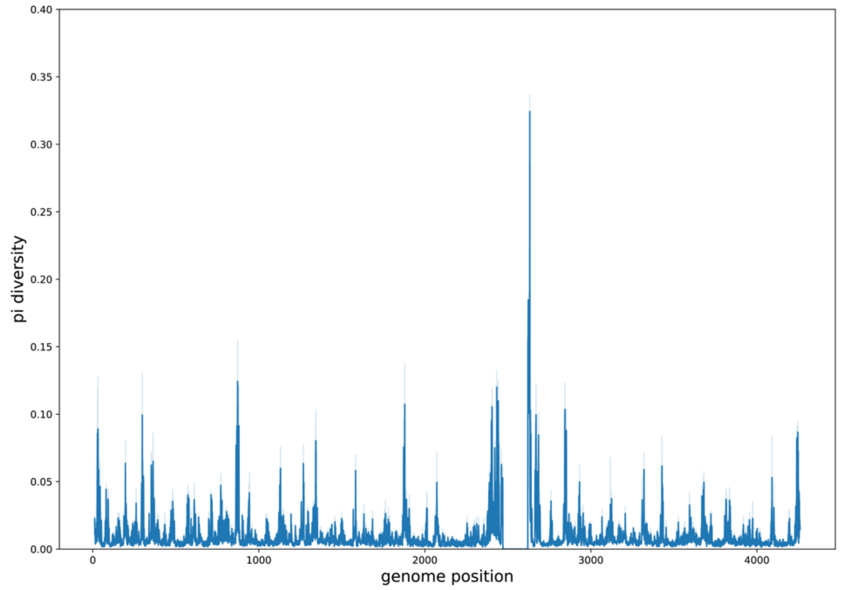

Figure S2. Nucleotide diversity (π) along the genome coordinate. Dark blue represents the mean line, and light blue areas represent confidence intervals. The mean of pi diversity along the genome within 18 samples varied between 0.001 and 0.32.
